# Negative association between free triiodothyronine level and contrast-induced acute kidney injury in patients undergoing primary percutaneous coronary intervention

**DOI:** 10.1186/s12882-019-1386-y

**Published:** 2019-06-03

**Authors:** Kai-Yang Lin, Sun-Ying Wang, Hui Jiang, Han-Chuan Chen, Zhi-Yong Wu, Yan-Song Guo, Peng-Li Zhu

**Affiliations:** Department of Cardiology, Fujian Provincial Hospital, Fujian Medical University, Fujian Cardiovascular Institute, Fujian Provincial Center for Geriatrics, Fuzhou, 350001 China

**Keywords:** Free triiodothyronine, Contrast-induced acute kidney injury, Primary percutaneous coronary intervention

## Abstract

**Background:**

A low FT3 level is significantly associated with a variety of kidney disease and acute myocardial infarction (AMI). However, it remains unclear whether low FT3 is associated with CI-AKI in patients who underwent pPCI.

**Methods:**

Single-center retrospective study evaluated 363 STEMI patients undergoing pPCI. Patients were classfied into 2 groups, low FT3 group (FT3 < 3.1 pmol/L) and normal FT3 group (FT3 ≥ 3.1 pmol/L);CI-AKI was defined as an increase in the serum creatinine levels of ≥50% or 0.3 mg/dL above the baseline level within 48 h after contrast medium exposure.

**Results:**

Overall, 80(22.0%) patients had low FT3, and 59(16.3%) patients developed CI-AKI. The incidence of CI-AKI and in-hospital mortality was significantly higher in patients with low FT3 than normal (31.3% vs 12.0%; 15.0% vs 3.2%, respectively, both *p* < 0.0001). Multivariate logistic regression analysis indicated that low FT3 was an independent predictor of CI-AKI (odds ratio [OR] = 2.62, 95%CI:1.35–5.07, *p* < 0.05). In addition, low FT3 was associated with an increased risk of all-cause mortality during a mean follow-up period of 20 months (hazard ratio [HR] = 2.54, 95%CI:1.15–5.60, *p* < 0.05).

**Conclusion:**

Low FT3 was associated with CI-AKI, short- and long-term mortality in STEMI patients after pPCI.

**Electronic supplementary material:**

The online version of this article (10.1186/s12882-019-1386-y) contains supplementary material, which is available to authorized users.

## Background

Contrast-induced acute kidney injury (CI-AKI) is a frequent complication after percutaneous coronary intervention (PCI), which is the third most common cause of acute kidney injury in hospitalized patients [1] and is strongly associated with the risk of dialysis, mortality, length of stay and costs [2, 3].Thus, timely identifying patients at high risk of CI-AKI and taking early prophylactic measures are critical.

Thyroid hormones has been generally considered to be associated with kidney for several years, which involved in the growth, development and electrolyte balance maintenance of the kidney [4–6]. Previous studies have found that the deterioration of renal function is followed by changes in the synthesis, secretion, metabolism and elimination of thyroid hormones [4, 7]. Nonthyroidal illness syndrome (NTIS) is a common manifestation in critically ill patients (including severe cardiac disease and kidney disease) without pre-existing thyroid disease. A decrease in total serum triiodothyronine (T3) and free triiodothyronine (FT3) without elevation of thyroid-stimulating hormone (TSH) is the most common form of NTIS, which was known as low T3 syndrome [8, 9]. Low f3 levels have been reported to be independent predictors of mortality in patients with myocardial infarction [10, 11] or kidney disease [12]. However, it is uncertain whether low FT3 level is related to CI-AKI in patients with ST-elevation myocardial infarction (STEMI) undergoing primary percutaneous coronary intervention (pPCI). Therefore, we aimed to focus on the relationship between low FT3 and CI-AKI in patients undergoing pPCI, and discuss the impact of low FT3 on short and long-term mortality.

## Methods

### Study population

This retrospective study was conducted at Fujian Provincial Hospital and Fujian Cardiovascular Institute in China between January 2012 and December 2016. Consecutive 639 patients with STEMI undergoing pPCI were enrolled. Patients with malignant tumor (*n* = 6), end-stage renal disease (estimated glomerular filtration rate [eGFR] < 15 mL/min/1.73m^2^(*n* = 2), with a history of thyroid diseases (*n* = 8), abnormal serum levels of thyrotropic stimulating hormone (TSH) (*n* = 50), lack of pre-procedural or post-procedural serum creatinine (SCr) (*n* = 46), lack of FT3 level (*n* = 164) were excluded. Finally, 363 patients undergoing pPCI were included in the analysis. The study protocol was approved by the ethics committee of Fujian Provincial Hospital (Fujian, China).

### Laboratory investigations, cardiac catheterization and medications

We applied electro-chemiluminescence immunoassay (Roche Diagnostics, COBAS E601, Mannheim, Germany) to quantify the concentrations of serum FT3, free thyroxine (FT4) and TSH. Our hospital’s clinical laboratory defined the normal reference interval of serum TSH, FT4 and FT3 as 0.27–4.20 mIU/L, 12.0–22.0 pmol/L, 3.1–6.8 pmol/L, respectively. SCr was measured at admission, 24 h and 48 h after pPCI. Echo-cardiography were applied to quantify the left ventricular ejection fraction (LVEF) during hospitalization. PCI procedures were performed by experienced interventional cardiologists through the femoral artery or radial artery approach according to standard techniques. Nonionic, low-osmolar contrast media (either Ultravist or Iopamiron, 370 mgI/mL) was used in all procedures. Medication therapy includs antiplatelet agents, anticoagulant, statins, β-blocking agents, angiotensin-converting enzyme inhibitors (ACEIs)/angiotensin receptor blocker (ARB), which were prescribed by cardiologists according to guidelines instructions. All patients received a standard hydration protocol which is administration of intravenous isotonic saline (0.9%) at a rate of 1 mL/kg/h for 12 h (or 0.5 mL/kg/h for 12 h if patients had overt heart failure).

### Definitions and follow-up

CI-AKI was considered the primary outcome and was defined as an SCr increase greater than 0.3 mg/dl or an increase greater than 50% within 48 h after contrast agent administration [13]. Secondary outcomes include long-term all-cause mortality and in-hospital mortality. Low FT3 was defined as a serum FT3 level below the lower limit of the reference interval (FT3 < 3.1 pmol/L [11, 14]) with normal TSH value. eGFR was calculated using CKD-EPI equation: eGFR = 141 × (minimum of standardized SCr [mg/dL]/ κ, 1)^α^ × (maximum of standardized SCr [mg/dL]/ κ, 1)^-1.209^ × 0.993 ^age^ ×(1.018 if female) × (1.159 if black), where κ is 0.7 for female and 0.9 for male and α is − 0.329 for female and − 0.411 for male [15]. The definition of peri-hypotension referred to a durative systolic blood pressure (SBP) beneath 80 mmHg for at least 1 h in which required notropic medications or required intra-aortic balloon pump (IABP) within 24 h before or after procedure [16]. A hematocrit (HCT) <0.39 (male) or < 0.36 (female) was considered anemia [16].

All patients were follow-up by trained nurses using either outpatient clinical visits or telephone.

### Statistical analysis

Analyses were performed using the SPSS software package (version 20.0). Data are described as the mean ± standard deviation (SD), medians and inter-quartile ranges, or percentages. Continuous variables were evaluated by Student’s t-test or Wilcoxon rank-sum test while categorical variables by Chi-square or Fisher exact test. Univariate and multivariate logistic regression analyses were used to identify the independent risk factors of CI-AKI and in-hospital mortality. Cox regression analysis was performed to evaluate independent predictors of long-term mortality, and the Kaplan-Meier curve was used to assess the survival time between different groups with a log-rank test. All statistical assessments were two-tailed, and significance was set as *p* < 0.05.

## Results

### Baseline characteristics

Among the 363 patients, 59(16.3%) patients developed CI-AKI. Subjects’ demographics and clinical characteristics between AKI group and No-AKI group are presented in Table 1.Patients with CI-AKI tended to be older, have higher rate of peri-procedural hypotension, use of IABP and receive a higher volume of contrast media. Patients with CI-AKI had significantly lower baseline FT3, LVEF, and higher baseline SCr level than those without CI-AKI. Patients with CI-AKI were also more likely to be treated with diuretics (Table 1).

**Table 1:** Baseline Clinical and Demographic Characteristics in Patients With and Without CI-AKI

Variables	Total	CI-AKI(−)	CI-AKI(+)	*P*-value
	(*n* = 363)	(*n* = 304)	(*n* = 59)	
Demographics				
Age, years	64.07 ± 12.52	63.39 ± 12.29	67.58 ± 13.18	0.019
Age > 75 years, n (%)	72 (19.8%)	52 (17.1%)	20 (33.9%)	0.003
Sex, female, n (%)	56 (15.4%)	47 (15.5%)	9 (15.3%)	0.968
Medical history				
Smoker	207 (57.0%)	173 (56.9%)	34 (57.6%)	0.919
Prior PCI, n (%)	9 (2.5%)	7 (2.3%)	2 (3.4%)	0.644
Prior myocardial infarction, n (%)	7 (1.9%)	5 (1.6%)	2 (3.4%)	0.318
Hypertension, n (%)	218 (60.1%)	181 (59.5%)	37 (62.7%)	0.649
Diabetes, n (%)	99 (27.3%)	79 (26.0%)	20 (33.9%)	0.212
Anemia, n (%)	103 (28.6%)	85 (28.2%)	18 (30.5%)	0.724
Laboratory measurements				
fT3, pmol/L	3.67 ± 0.86	3.74 ± 0.83	3.33 ± 0.94	0.001
fT4, pmol/L	16.06 ± 3.70	15.91 ± 3.81	16.82 ± 2.95	0.085
TSH, mIU/L	0.77 (0.49–1.31)	0.77 (0.50–1.26)	0.79 (0.48–1.59)	0.770
Low fT3, n (%)	80 (22.0%)	55 (18.1%)	25 (42.4%)	<0.0001
Serum creatinine, mg/dl	0.81 (0.69–0.97)	0.80 (0.68–0.95)	0.86 (0.75–0.86)	0.027
Serum creatinine>1.5 mg/dl, n (%)	15 (3.9%)	6 (2.0%)	9 (15.3%)	<0.0001
eGFR, mL/min per 1.73 m^2^(MDRD)	98.23 (81.09–118.69)	99.65 (82.35–119.51)	89.17 (67.62–110.62)	0.014
eGFR< 60 mL/min per 1.73 m^2^(MDRD)	29 (8.0%)	18 (5.9%)	11 (18.6%)	0.001
eGFR, mL/min per 1.73 m^2^(CKD-EPI)	91.21 (78.25–101.78)	93.78 (79.57–102.11)	84.83 (61.67–94.93)	0.002
eGFR< 60 mL/min per 1.73 m^2^(CKD-EPI)	34 (9.4%)	21 (6.9%)	13 (22.0%)	<0.0001
Hemoglobin, g/l	139.30 ± 17.33	139.83 ± 16.12	136.59 ± 22.47	0.190
Hematocrit	0.41 ± 0.05	0.41 ± 0.04	0.4 ± 0.06	0.691
Cholesterol, mmol/l	4.84 ± 1.21	4.88 ± 1.20	4.63 ± 1.24	0.149
Triglyceride, mmol/l	1.25 (0.90–1.70)	1.28 (0.91–1.72)	1.14 (0.85–1.55)	0.08
LVEF,%	52.82 ± 8.38	53.83 ± 7.67	47.04 ± 9.90	<0.0001
LVEF < 0.45, n (%)	66 (18.2%)	44 (14.5%)	22 (37.3%)	<0.0001
Medication, n (%)				
Antiplatelet, n (%)	355 (97.8%)	298 (98.0%)	57 (96.6%)	0.501
Statin, n (%)	362 (99.7%)	303 (99.7%)	59 (100.0%)	0.659
Diuretic, n (%)	205 (56.5%)	158 (52.0%)	47 (79.7%)	<0.0001
Procedure performed				
Number of diseased vessels, n	1.93 ± 1.08	1.88 ± 1.10	2.19 ± 0.92	0.481
Stent length	33 (23–51)	28 (23–51)	33 (23–47)	0.305
Perioperative hypotension, n (%)	130 (35.8%)	95 (31.2%)	35 (59.3%)	<0.0001
IABP, n (%)	12 (3.3%)	5 (1.6%)	7 (11.9%)	<0.0001
Contrast volume > 200 ml, n (%	270 (74.4%)	219 (72.0%)	51 (86.4%)	0.020

Abbreviations:*CI-AKI* contrast-induced acute kidney injury, *PCI* percutaneous coronary intervention, *fT*3 free triiodothyronine, *fT*4 free thyroxine, *TSH* thyroid stimulating hormone, *eGFR* estimated glomerular filtration rate, *LVEF* left ventricular ejection fraction, *IABP* intra-aortic balloon pump

### In-hospital outcomes between low FT3 level and normal

The incidences of CI-AKI, in-hospital mortality and stent thrombosis were significantly higher in patients with low FT3 than normal (31.3% vs 12.0%,15.0% vs 3.2, 2.5% vs 0%, all *p* < 0.05). Patients with CI-AKI had significantly higher risk of in-hospital mortality, required RRT and bleeding (22.0% vs 2.6%,6.8% vs 1.3, 8.5% vs 0.7%, all *p* < 0.05) (Table 2).

**Table 2:** Incidence of In-Hospital Outcomes

Outcome	Normal fT3	Low fT3	*P*-value	CI-AKI(−)	CI-AKI(+)	*P*-value
	(*n* = 283)	(*n* = 80)		(*n* = 304)	(*n* = 59)	
CI-AKI	34 (12.0%)	25 (31.3%)	<0.0001			
In-hospital mortality, n (%)	9 (3.2%)	12 (15.0%)	<0.0001	8 (2.6%)	13 (22.0%)	<0.0001
Recurrent MI, n (%)	2 (0.7%)	2 (2.5%)	0.212	3 (1.0%)	1 (1.7%)	0.510
Required RRT, n (%)	4 (1.4%)	4 (5.0%)	0.075	4 (1.3%)	4 (6.8%)	0.027
Stent thrombosis, n (%)	0 (0.0%)	2 (2.5%)	0.048	1 (0.3%)	1 (1.7%)	0.299
Bleeding, n (%)	5 (1.8%)	2 (2.5%)	0.652	2 (0.7%)	5 (8.5%)	0.002

Abbreviations:*CI-AKI* contrast-induced acute kidney injury, *fT*3 free triiodothyronine, *RRT* Renal replacement therapy, *MI* Mycardial infarction

### Association between low FT3 level and CI-AKI

**Fig. 1:**
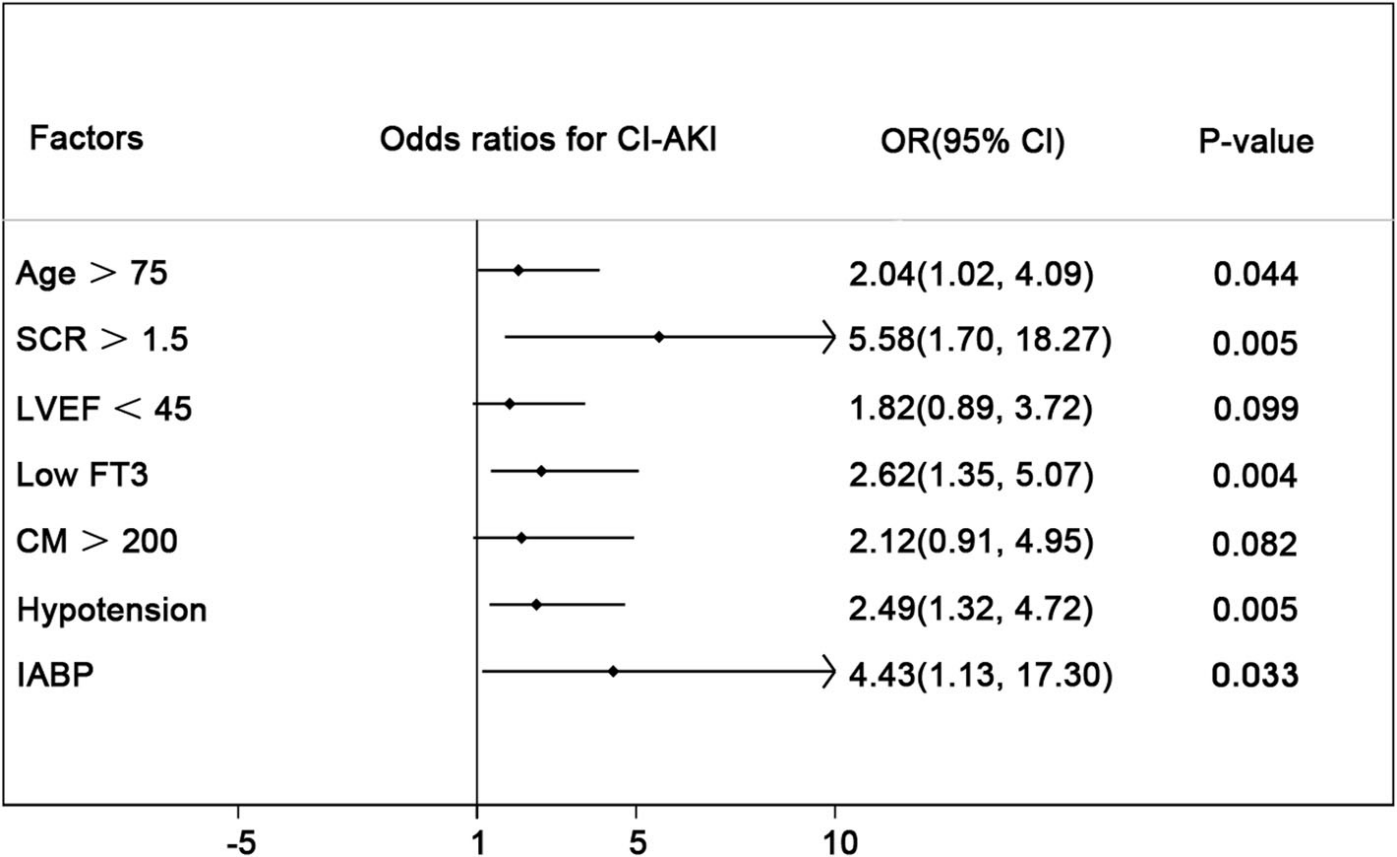
Multivariate logistic analysis for CI-AKI

Seven of the risk factors include age > 75 years, SCr baseline> 1.5 mg/dL, LVEF <45%, use of contrast volume > 200 mL, use of IABP, peri-procedural hypotension and low FT3 were associated with CI-AKI in univariate logistic analysis (all *p* < 0.05)(Additional file 1: Table S1). Multivariate analysis indicates that age > 75 years(OR = 2.04, 95%CI:1.02–4.09, *p* = 0.044), SCr > 1.5 mg/dl (OR = 5.58, 95%CI:1.70–18.27, *p* = 0.005), peri-procedural hypotension (OR = 2.49, 95%CI:1.32–4.72, *p* = 0.005), IABP use (OR = 4.43, 95%CI:1.13–17.30, *p* = 0.033) and low FT3(OR = 2.62,95%CI:1.35–5.07, *p* = 0.004) were independent risk factors of CI-AKI in patients after pPCI (Fig. 1).

### Association between low FT3 level and long-term mortality

**Fig. 2:**
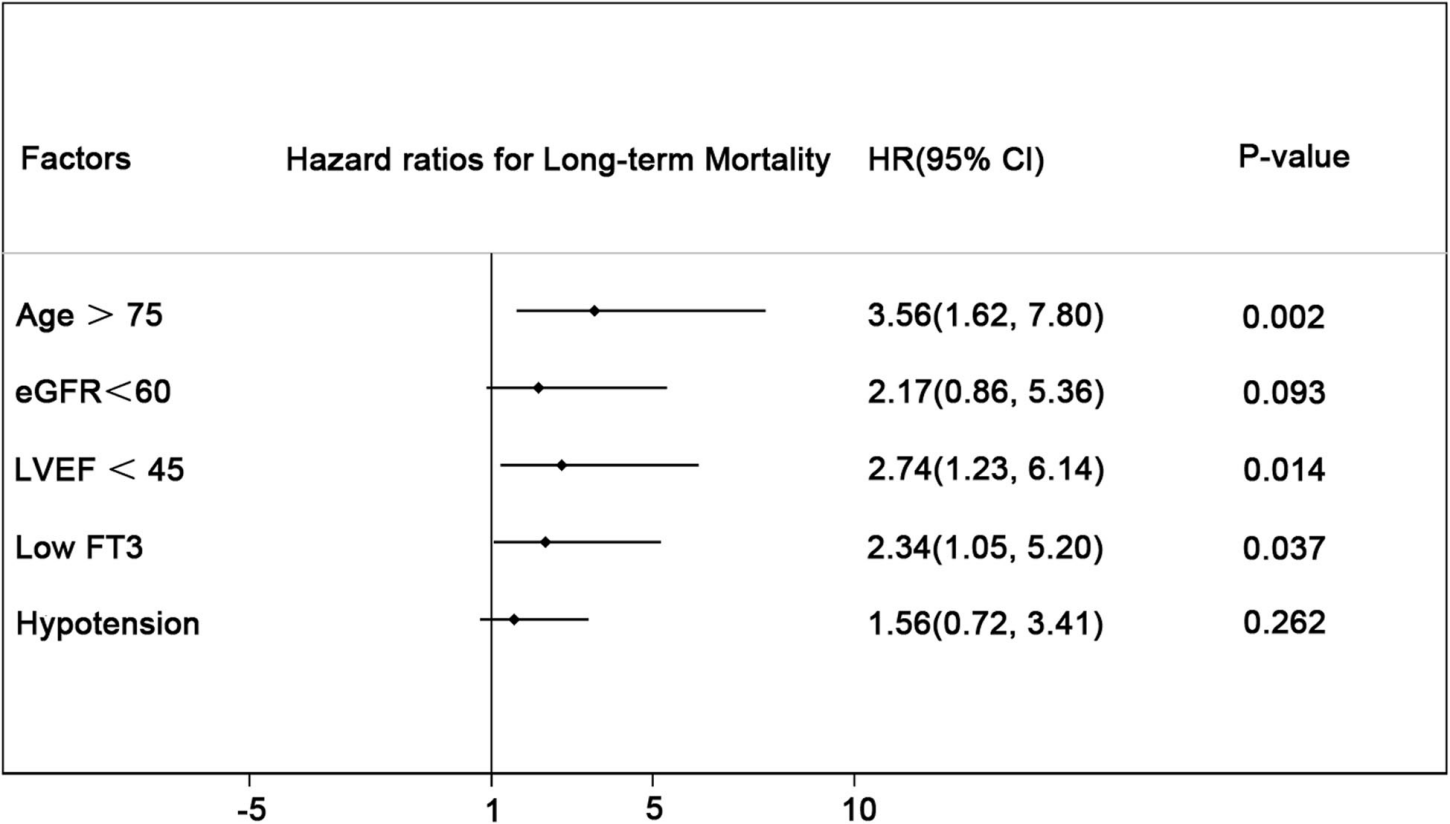
Cox regression analysis for long-term mortality

The mean follow-up period was 20 months. Cox regression analysis showed that low FT3 was an independent risk factor for long-term mortality (HR = 2.54, 95%CI:1.15–5.60, *p* < 0.05) after adjusting for other risk factors including age > 75 years, eGFR< 60 mL/min per 1.73 m^2^, LEVF <45% and peri-procedural hypotension (Fig. 2).

**Fig. 3:**
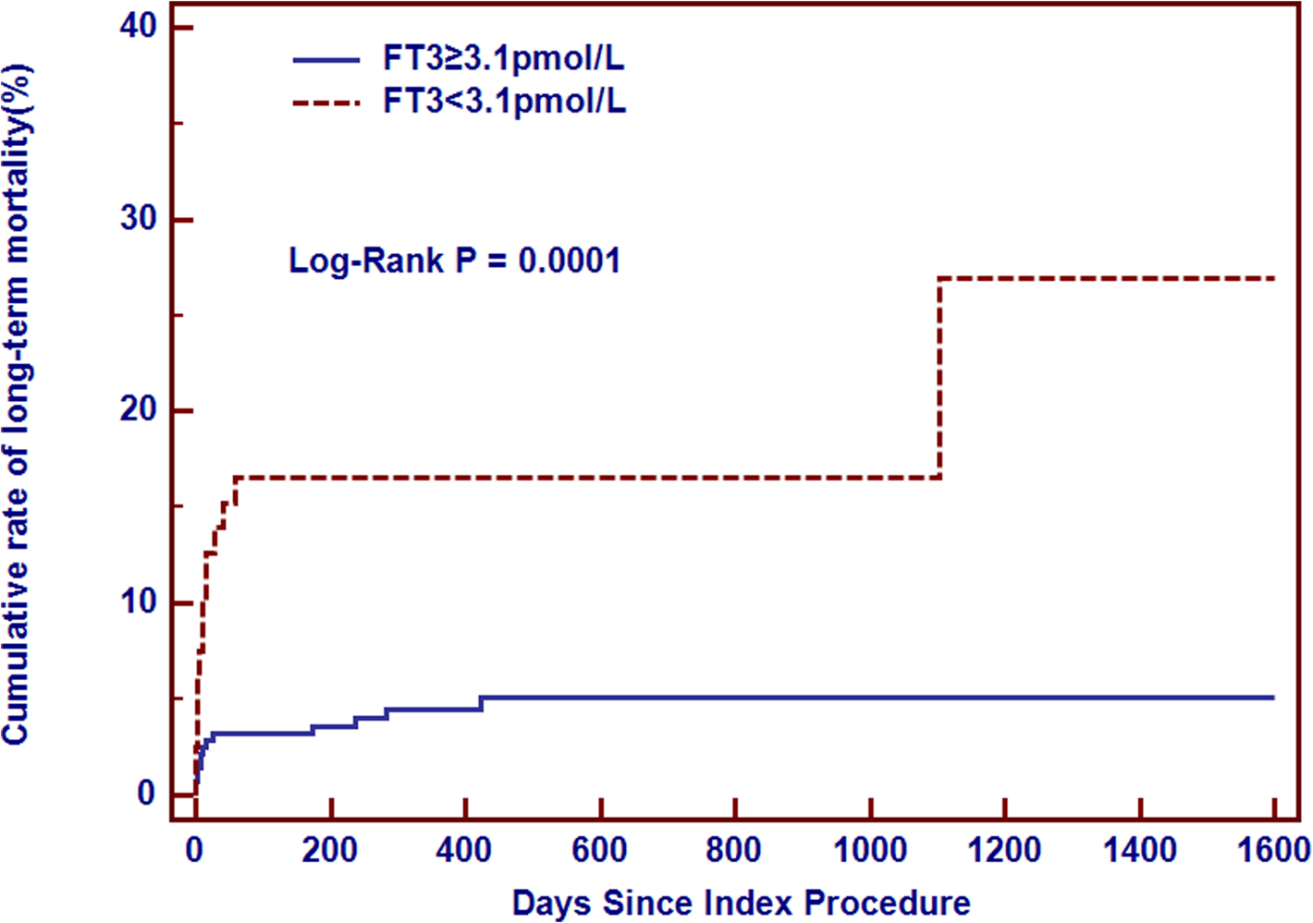
Cumulative rate of mortality between patients with low fT3 and normal

The Kaplan-Meier curve demonstrated that low FT3 group had higher rate of long-term mortality than normal FT3 group (Chi-Square = 15.60, Log-Rank *p* = 0.0001) (Fig. 3).

## Discussion

The relationship between low T3 syndrome or thyroid dysfunction and AKI has been reported previously [17, 18], yet the relationship between low FT3 and CI-AKI has rarely been investigated. In this study we found that a serum FT3<3.1 pmol/L was an independent risk factor of CI-AKI, as well as a predictor of long-term mortality after pPCI.

The incidence of CI-AKI after pPCI ranges from 11.9 to 17.1% according to previous studies, which is significantly higher than that of elective PCI [19–23]. As with above studies, our study also demonstrated a similar high incidence of 15% in this population, which is strongly associated with blood volume insufficiency, hemodynamic instability and inadequate hydration. Meanwhile, CI-AKI has been considered to be associated with a poor prognosis. Thus it’s crucial to screen the risk factors of CI-AKI in these patients and to initiate early preventive measures.

Previous studies had identified several risk factors for predicting CI-AKI after pPCI, such as pre-existing renal impairment, advanced age, high contrast medium volume use of intra-aortic balloon pump (IABP), peri-procedural hypotension, and hyperuricemia [16, 19, 23–26]*,* part of which were also identified as risk factors of CI-AKI in our study. Furthermore, we also found low FT3 was a novel risk factor of CI-AKI after pPCI.

Low FT3 levels are common in patients with chronic kidney disease (CKD). According to the study by Kaptein et al. [7], low FT3 occured in 66% of the 287 euthyroid patients with end-stage renal disease (eGFR< 15 mL/min·1.73 m^2^). In addition, Sang Heon Song et al. [27] demonstrated the increasing incidence of low T3 along with the increase of CKD stage, even after adjusting for confounders, eGFR remained positively related to T3 levels. There are also growing evidence suggesting low FT3 exerts dramatic effects upon kidney function in various aspects. A more recent study [28] including 2180 euthyroid participants revealed that serum FT3 levels were positively related to both eGFR and creatinine clearance (CrCl) but fT4 and TSH had no significant correlation with either eGFR or CrCl. In an another study [29], the incidence of low FT3 was 71.7% in 669 dialysis patients, low T3 is not only closely associated with CKD but also significantly increases the risk of poor outcomes in CKD patients, furthermore, low FT3 was found to be an independent predictor of cardiovascular mortality (HR = 1.75,95%CI: 1.19–2.57, *p* = 0.005) in hemodialysis patients during a mean follow-up period of 34 ± 16 months. A cohort study [30] including 114 dialysis patients reinforced that patients with low FT3 had higher risk of all-cause mortality and a shorter survival time than the patients with normal FT3. Although above findings provided a comprehensive view that low FT3 was associated with CKD and was a significant predictor for mortality.

Several studies have also suggested that thyroid function disorders including low FT3 are correlated to AKI. In a prospective study by Iglesias P et al. [17] which enrolled 35 consecutive patients with AKI, alteration in thyroid function tests was detected in over 80% of these patients and the most common disorder was low T3 syndrome. However, due to its short follow-up period and small sample size, this study failed to determine the prognostic value of thyroid function tests for AKI. Another study by Zhang D et al. [18] including 1339 patients in the general intensive care units demonstrated that when Cystatin C (Cys C) was used for detecting early AKI, FT3 was found to be independently associated with Cys C. Furthermore, FT3 had no significant impact on the diagnostic and predictive accuracy of Cys C in detecting AKI in ICU patients. A more recent study by us firstly investigated the relationship between FT3 concentration and CI-AKI in patients aged > 75 years undergoing PCI, which showed the incidence of CI-AKI was about 1.4 folds higher in patients with low FT3 compared with normal FT3, suggesting low FT3 might be a novel risk factor for CI-AKI [31]. It’s well-known that low FT3 is more frequent in critical ill patients such as myocardial infarction. However, the relationship between FT3 and CI-AKI in patients undergoing pPCI is unclear, our findings of the present study fills the gap by demonstrating that FT3 < 3.1 pmol/L was a powerful predictor for CI-AKI in patients after pPCI, even after adjusting for potential confounding factors.

The underlying mechanism has not yet been fully elucidated, several plausible explanations are stated as follows. First, as stated above, low FT3 may be an marker reflecting severe cardiac damage, in which extensive cardiac damage had greater impairment of cardiac function, further declining cardiac output and reducing renal blood flow, and deteriorated renal function [32]. Second, low FT3 level was also considered to be a maladaptive neurohumoral alteration during acute ill condition, for that lower FT3 level contributed to the preservation of energy yet in turn weakened the regeneration and self-repair capability of tubular epithelium [29, 33]. Third, previous studies confirmed that low FT3 directly led to the contraction of renal artery through impaired endothelial function in vitro and in vivo, and deteriorated kidney function [34, 35]. Finaly, the decline of FT3 levels may be caused by decreased conversion of the prohormone T4 into T3 and by increased T3 catabolism, they were associated with hypoxia, inflammation, and oxidative stress, which also be involved in the process of CI-AKI [36–38].

A low FT3 level is also a common phenomenon reflecting severe cardiac damage and impairment of cardiac function, so that increasing the risk of mortality and other poor outcomes. A recent meta-analysis [39] including 41 studies showed that the incidence of low FT3 in patients with acute myocardial infarction up to 18.9%. Another retrospective study [10] including 501 STEMI patients in China found a even higher rate (34.1%) of low FT3 levels in AMI patients, the low FT3 group had a higher serum levels of TnT, NT-ProBNP, and higher percentage of three diseased vessels, which indicate more serious myocardial injury, furthermore, a low FT3 level was found to be independently associated with short-and long-term death and MACE. Another more recent study by Y Song et al. [11] in 699 consecutive STEMI patients also found that low FT3 levels were independently associated with 30-day and 1-year all-cause death and MACE. Conversely, another prospective study [40] include 457 STEMI patients undergoing pPCI showed that serum FT3 levels were associated with in-hospital MACE and long-term MACE only in univariate analysis but not in multivariate analysis, however, in this study the relationship among risk factors and long-term MACE were analyzed by multivariate logistic regression analyses but not cox regression analyses, which does not consider the time-event factor. In our study, poor prognosis for long-term mortality in STEMI patients with low FT3 undergoing pPCI was also observed. Compared with patients with normal FT3, patients with low FT3 had significantly higher incidence of long-term mortality during 20 months of follow-up (HR = 2.54, 95%CI:1.15–5.60, *p* < 0.05).

Our study also has several limitations. First, due to the single-center, retrospective nature of this study and the small population included, there may be residual confounding by unmeasured factors. Second, since some parameters were not routinely tested in our hospital, we failed to quantify these parameters including serum reverse T3 in our study. Third, there is a possibility that the true incidence of CI-AKI may have been underestimated in clinical practice due to the potential loss of real peak SCR levels which is restrained by measurement times. Fourth, we failed to explored the optimal cutoff of fT3 concentration for predicting CI-AKI and mortality. Finally, it’s unable to explored the causal correlation between low fT3 and CI-AKI due to the retrospective design of this study, whether this association only simply reflects the severity of the disease of this population was beyond explanation.

## Conclusions

In summary, The study showed that serum concentration FT3 < 3.1 pmol/L was an independent risk factor for CI-AKI and long-term mortality in patients undergoing pPCI. Measurement of serum FT3 may help identify patients who were at high risk of CI-AKI and mortality after pPCI.

## Additional file

Additional file 1: **Table S1.** Univariate Logistic Analysis Associating CI-AKI Risk Fascors. (DOCX 13 kb)

## Abbreviations

CI-AKI: contrast-induced acute kidney injury
eGFR: estimated glomerular filtration rate
FT3: free triiodothyronine
fT4: free thyroxine
IABP: intra-aortic balloon pump
LVEF: left ventricular ejection fraction
PCI: percutaneous coronary intervention
SCr: serum creatinine
TSH: thyroid stimulating hormone
