# Supplementary material for: Negative association between free triiodothyronine level and contrast-induced acute kidney injury in patients undergoing primary percutaneous coronary intervention
Source: BMC Nephrol. 2019 Jun 3;20:201. doi: 10.1186/s12882-019-1386-y (PMC6545736; doi:10.1186/s12882-019-1386-y)
Supplement: Supplementary file 1 — Table S1. Univariate Logistic Analysis Associating CI-AKI Risk Fascors. (DOCX 13 kb) [file 12882_2019_1386_MOESM1_ESM.docx]

**Supplement Table1 Univariate Logistic Analysis Associating CI-AKI Risk Fascors**

| Risk factors | Univariate logistic regression | | |
| --- | --- | --- | --- |
|  | OR | 95% CI | *P*-value |
| Age>75ys | 2.485 | 1.342-4.602 | 0. 004 |
| Serum creatinine>1.5mg/dl | 8.940 | 3. 050-26.208 | <0.0001 |
| LVEF < 45% | 3.514 | 1.896-6.511 | <0.0001 |
| fT3 <3.1pmol/l | 3.329 | 1.839-6.025 | <0.0001 |
| Contrast volume > 200ml | 2.474 | 1.127-5.432 | 0. 024 |
| Peri-procedural hypotension | 3.208 | 1.808-5.692 | <0.0001 |
| Use of IABP | 8.050 | 2.462-26.324 | <0.0001 |

Abbreviations:CI-AKI: contrast-induced acute kidney injury; OR: odds ratio; CI:confidence interval; fT3:free triiodothyronine; IABP:intra-aortic balloon pump.

.
